# Hydrophobicity‐enhanced ferritin nanoparticles for efficient encapsulation and targeted delivery of hydrophobic drugs to tumor cells

**DOI:** 10.1002/pro.4819

**Published:** 2023-12-01

**Authors:** Alessio Incocciati, Jan Kubeš, Roberta Piacentini, Chiara Cappelletti, Sofia Botta, Lucia Bertuccini, Tomáš Šimůnek, Alberto Boffi, Alberto Macone, Alessandra Bonamore

**Affiliations:** ^1^ Department of Biochemical Sciences “A. Rossi Fanelli” Sapienza University of Rome Rome Italy; ^2^ Department of Biochemical Sciences, Faculty of Pharmacy in Hradec Králové Charles University Hradec Králové Czech Republic; ^3^ Center of Life Nano‐ and Neuro‐Science Italian Institute of Technology Rome Italy; ^4^ Core Facilities, Istituto Superiore di Sanità Rome Italy

**Keywords:** doxorubicin, drug delivery, ellipticine, ferritin, hydrophobic drugs, nanoparticle, protein engineering, tumor cells

## Abstract

Ferritin, a naturally occurring iron storage protein, has gained significant attention as a drug delivery platform due to its inherent biocompatibility and capacity to encapsulate therapeutic agents. In this study, we successfully genetically engineered human H ferritin by incorporating 4 or 6 tryptophan residues per subunit, strategically oriented towards the inner cavity of the nanoparticle. This modification aimed to enhance the encapsulation of hydrophobic drugs into the ferritin cage. Comprehensive characterization of the mutants revealed that only the variant carrying four tryptophan substitutions per subunit retained the ability to disassemble and reassemble properly. As a proof of concept, we evaluated the loading capacity of this mutant with ellipticine, a natural hydrophobic indole alkaloid with multimodal anticancer activity. Our data demonstrated that this specific mutant exhibited significantly higher efficiency in loading ellipticine compared to human H ferritin. Furthermore, to evaluate the versatility of this hydrophobicity‐enhanced ferritin nanoparticle as a drug carrier, we conducted a comparative study by also encapsulating doxorubicin, a commonly used anticancer drug. Subsequently, we tested both ellipticine and doxorubicin‐loaded nanoparticles on a promyelocytic leukemia cell line, demonstrating efficient uptake by these cells and resulting in the expected cytotoxic effect.

## 1 INTRODUCTION

Protein nanoparticles have emerged as a promising class of drug delivery systems, addressing the limitations of conventional approaches. These nanoparticles possess unique structural properties, biocompatibility, and biodegradability, making them highly attractive for therapeutic applications (Kianfar, 2021; Hong et al., 2020). Their sizes typically range from a few nanometers to several hundred nanometers, and they can be produced through various methods, including self‐assembly, coacervation, and protein engineering, each imparting distinct properties and functionalities (Habibi et al., 2022; Miao et al., 2022; Abdelkhaliq et al., 2018). One of the key advantages of protein nanoparticles is their ability to encapsulate a wide range of hydrophobic and hydrophilic compounds, nucleic acids, and proteins (Boisguérin et al., 2021; Segel et al., 2021; Olshefsky et al., 2022; Calisti et al., 2018). These nanoparticles exhibit high drug‐loading capacity and controlled release profiles, thus safeguarding the encapsulated drug from degradation and enhancing its solubility and stability. In addition, they offer the flexibility to incorporate targeting ligands or functional groups, enabling enhanced specificity and selectivity towards specific cell types or tissues (Spicer et al., 2018).

Ferritins, well‐characterized proteins involved in iron storage and homeostasis, are actually considered as promising protein nanocages for drug delivery applications (Song et al., 2021; He et al., 2019). In particular, human ferritin displays a key feature that consists in its capability to target specifically the transferrin receptor TfR1, overexpressed on the surface of most cancer cells (Daniels et al., 2012). From the structural point of view, ferritins are composed of 24 subunits that assemble into a hollow sphere with an 8 nm internal cavity. Such compact structure is extremely stable from the thermodynamic point of view but can be readily disassembled and reassembled through pH changes, facilitating the encapsulation of the desired cargo (Kim et al., 2011; Inoue et al., 2021). The internal cavity of ferritin is an ideal cargo space for drug delivery as it can be genetically or chemically modified to accommodate various types of molecules or drugs, including hydrophobic compounds, and positively/negatively charged molecules (Zhang et al., 2021; Wang et al., 2022; Incocciati et al., 2022; Ning et al., 2023). For example, these modifications allowed the loading of small interfering RNA (siRNA) (Li et al., 2016; Palombarini et al., 2021), basic therapeutic proteins (Macone et al., 2019; Palombarini et al., 2020), and conventional antitumor drugs (Sun et al., 2021) into the protein cage.

The internal surface of ferritin is primarily composed of hydrophilic residues, which can restrict the encapsulation efficiency of hydrophobic molecules (Jiang et al., 2020). Many of these hydrophobic molecules have a high efficacy profile in treating various pathologies, but their use is hindered by their poor solubility (Wu et al., 2011). Therefore, it is worth considering modifying the internal surface of ferritin to enhance its hydrophobic properties. For instance, Z. Wang et al. have engineered the inner surface of ferritin by introducing hydrophobic peptides of varying lengths (ranging from 6 to 22 amino acids) at the C‐terminus (Wang et al., 2022). This modification demonstrated that this new protein can effectively encapsulate hydrophobic drugs. However, it is important to note that despite the increased hydrophobicity, the length of the peptides can limit the available space within the inner cavity, consequently affecting the number of hydrophobic molecules that can be encapsulated.

In this study, we chose a distinct strategy to enhance the hydrophobic nature of ferritin's internal surface. Through mutagenesis techniques we replaced specific amino acids (4–6 residues per ferritin subunit) with tryptophan residues. This modification aimed to enhance the inner surface's hydrophobicity while preserving the protein's overall structure without impacting the inner volume. To demonstrate the potential of the modified ferritins here described, we used ellipticine (5,11‐dimethyl‐6H‐pyrido[4,3‐b] carbazole), a hydrophobic fluorescent natural alkaloid known for its multimodal activity against various types of cancer (Stiborová et al., 2011). Despite its interesting properties, the hydrophobic nature and significant side effects of ellipticine limit its clinical use. Thus far, in order to increase the bioavailability of ellipticine, it becomes crucial to employ suitable delivery systems that enhance its stability in aqueous solutions and reduce its systemic toxicity (Dan et al., 2020). Over the years, several approaches have been developed to enhance the solubility and targeted delivery of ellipticine (Tesarova et al., 2020; Wu et al., 2012; Abdulwahid et al., 2023; Studenovský et al., 2015). These approaches involve encapsulating ellipticine within nanoparticles of various compositions, potentially decorated on the surface to impart selectivity. Our work is in line with this context, since we encapsulated ellipticine within a ferritin mutant with a hydrophobic cavity, which exhibited superior efficiency compared to that of the wild‐type ferritin. The efficacy of this hydrophobicity‐enhanced ferritin nanoparticle was thus evaluated on a promyelocytic leukemia cell line expressing the TfR1 receptor.

## 2 RESULTS AND DISCUSSION

### 2.1 Protein nanoparticle characterization

**FIGURE 1:**
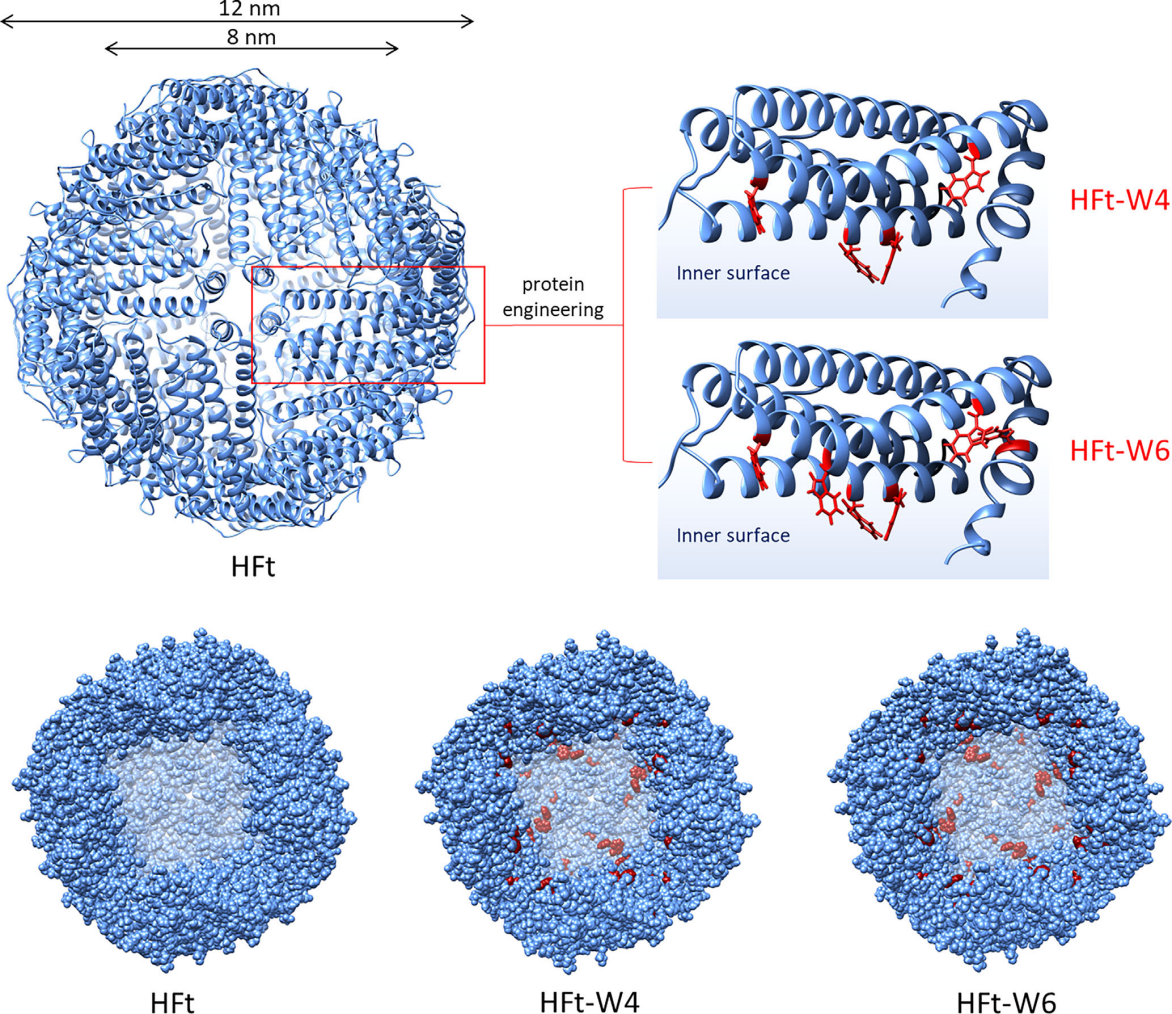
Comparison of human H ferritin and hydrophobicity‐enhanced HFt‐W4 and HFt‐W6 mutants. The diagram illustrates the structural representation of human H ferritin and its mutants, HFt‐W4 and HFt‐W6. Within each subunit, four (in HFt‐W4) or six (in HFt‐W6) amino acid residues were replaced with tryptophan residues (highlighted in red), resulting in the respective mutants HFt‐W4 and HFt‐W6. The tryptophan substitutions were strategically positioned to point towards the internal cavity of the nanoparticle, enhancing its hydrophobicity for improved drug encapsulation.

The aim of this study was to develop a ferritin‐based protein nanoparticle suitable for the targeted delivery of hydrophobic drugs. To achieve this goal, we employed a rational protein engineering approach to modify the internal cavity of human H ferritin (HFt). In particular, we replaced histidine, phenylalanine, and tyrosine residues whose side chains are exposed to the internal cavity with tryptophan residues on each subunit, resulting in the modification of 4 or 6 amino acids per subunit (Figure 1). These residues (HFt‐W4: H58W, H61W, H129W, H152W; HFt‐W6: H58W, H61W, H129W, Y138W, H152W, F171W) were carefully selected from those not involved in ferroxidase activity, in stabilizing the protein structure, and facing the interior of the cavity.

The resulting 24‐mer structure of ferritin displayed a substantially modified polarity of the internal cavity, which can accommodate a variety of hydrophobic drugs. This modification was achieved with only a small number of amino acid changes, which is advantageous for preserving the stability and integrity of the protein nanoparticle.

**FIGURE 2:**
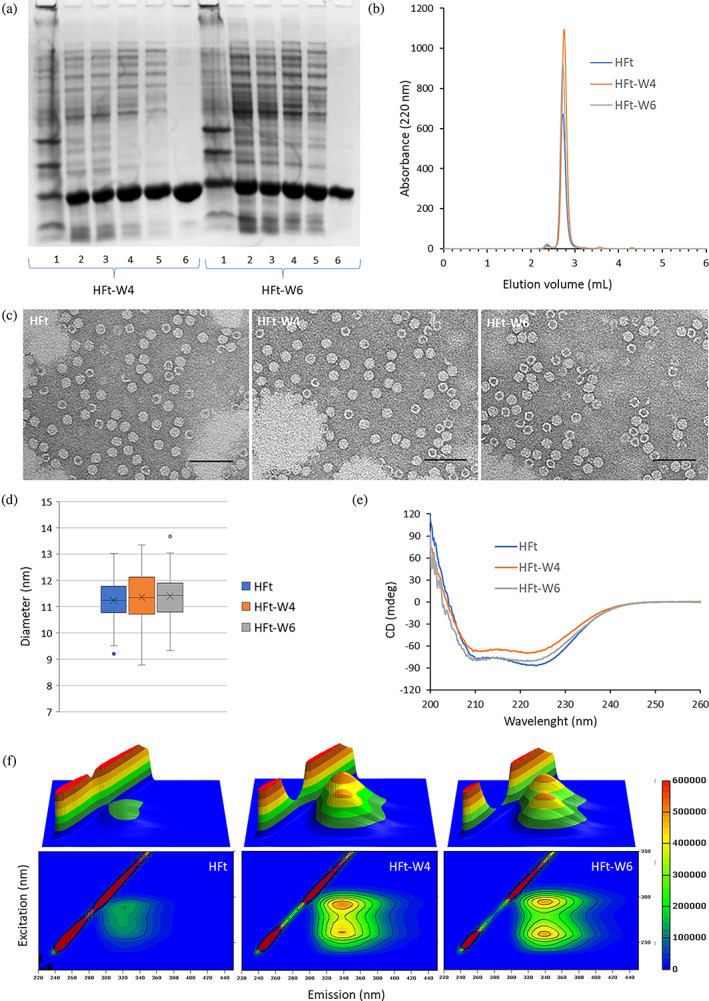
Characterization of HFt‐W4 and HFt‐W6 mutants. (a) SDS‐PAGE of HFt‐W4 and HFt‐W6 (1: marker; 2: sonication supernatant; 3: 70% ammonium sulfate pellet; 4: heat treatment (70°C); 5: Post‐DNAse treatment; 6: post gel filtration). (b) HP‐SEC chromatograms of human H ferritin, HFt‐W4 and HFt‐W6. (c) TEM negative staining of HFt, HFt‐W4, and HFt‐W6 at a protein dilution of 15 μg mL^−1^ (scale bar: 0.1 μm). (d) HFt, HFt‐W4 and HFt‐W6 diameter distributions measured by TEM image analysis. (e) CD spectra of HFt, HFt‐W4 and HFt‐W6. (f) Fluorescence 3D and contour maps of HFt, HFt‐W4 and HFt‐W6 (excitation: 220–350 nm; emission: 220–450 nm).

The HFt‐W4 and HFt‐W6 mutants were successfully expressed in soluble form and in high yields in *E. coli* and were highly purified using standard protocols involving fractional precipitation and size exclusion chromatography. The final purified ferritin nanoparticles were characterized using SDS‐PAGE, high performance‐size exclusion chromatography (HP‐SEC), transmission electron microscopy (TEM), circular dichroism (CD), and fluorescence spectroscopy (Figure 2).

The results of the SDS‐PAGE analysis (Figure 2a) demonstrate that the two mutants were obtained in a highly purified form following the fractionation and molecular filtration steps. The high purity (accounting up to 99%) was further confirmed by the HP‐SEC analysis (Figure 2b), revealing that HFt‐W4 and HFt‐W6 mutants eluted as a single peak at the elution volume of wild‐type HFt. This observation provides evidence supporting the correct assembly of the ferritins in their 24‐mer structure. This was further confirmed by TEM analysis in which HFt and HFt‐W4 and HFt‐W6 mutants show the typical donut shape with the same average diameter of 11.5 nm (Figure 2c,d). In addition, CD analysis shows that the introduction of tryptophan residues on the inner surface of the protein cage does not alter its α‐helix secondary structure, as evidenced by the presence of double minima around 209 and 222 nm (Figure 2e). It is worth mentioning that slight differences in the shape of the CD spectra between HFt and the hydrophobicity‐enhanced mutants are likely due to the contribution of tryptophan absorption in the far‐UV region (Freskgaard et al., 1994).

Given the 24‐mer structure of ferritin, the HFt‐W4 and HFt‐W6 mutants contain additional 96 and 144 tryptophan residues respectively, as compared to the wild‐type ferritin. The presence of these additional tryptophans also provides an opportunity for convenient characterization of the mutants using fluorescence analysis.

In support of our characterization, Figure 2f presents the 3D fluorescence spectra obtained by UV excitation of the tryptophan residues within the mutants and wild‐type ferritin. These spectra clearly demonstrate a significant increase in the emission intensity of HFt‐W4 and HFt‐W6 compared to the HFt. The strong correlation between the increased emission intensity and the abundance of tryptophans in HFt‐W4 and HFt‐W6 provides further confirmation of the successful introduction of additional hydrophobic residues, such as aromatic indole moieties, in the inner surface of the protein cavity. It is important to underline that the mutations introduced do not affect the ferroxidase site of ferritin. In fact, the kinetics of iron oxidation confirm that the hydrophobicity‐enhanced mutants maintain their catalytic activity (Figure S1).

The HFt‐W4 and HFt‐W6 mutants, characterized by a greater hydrophobicity in the internal cavity were designed to encapsulate hydrophobic molecules. For this reason, it is crucial to evaluate their ability to disassemble and reassemble under the conditions typically used for the wild‐type protein. The most used method for this purpose is the pH jump technique: the protein is first dissociated by quickly lowering the pH to approximately 2.5 followed by gradual raising towards neutrality, a procedure that allows the 24‐mer to reassociate and encapsulate target molecules present in the solution.

**FIGURE 3:**
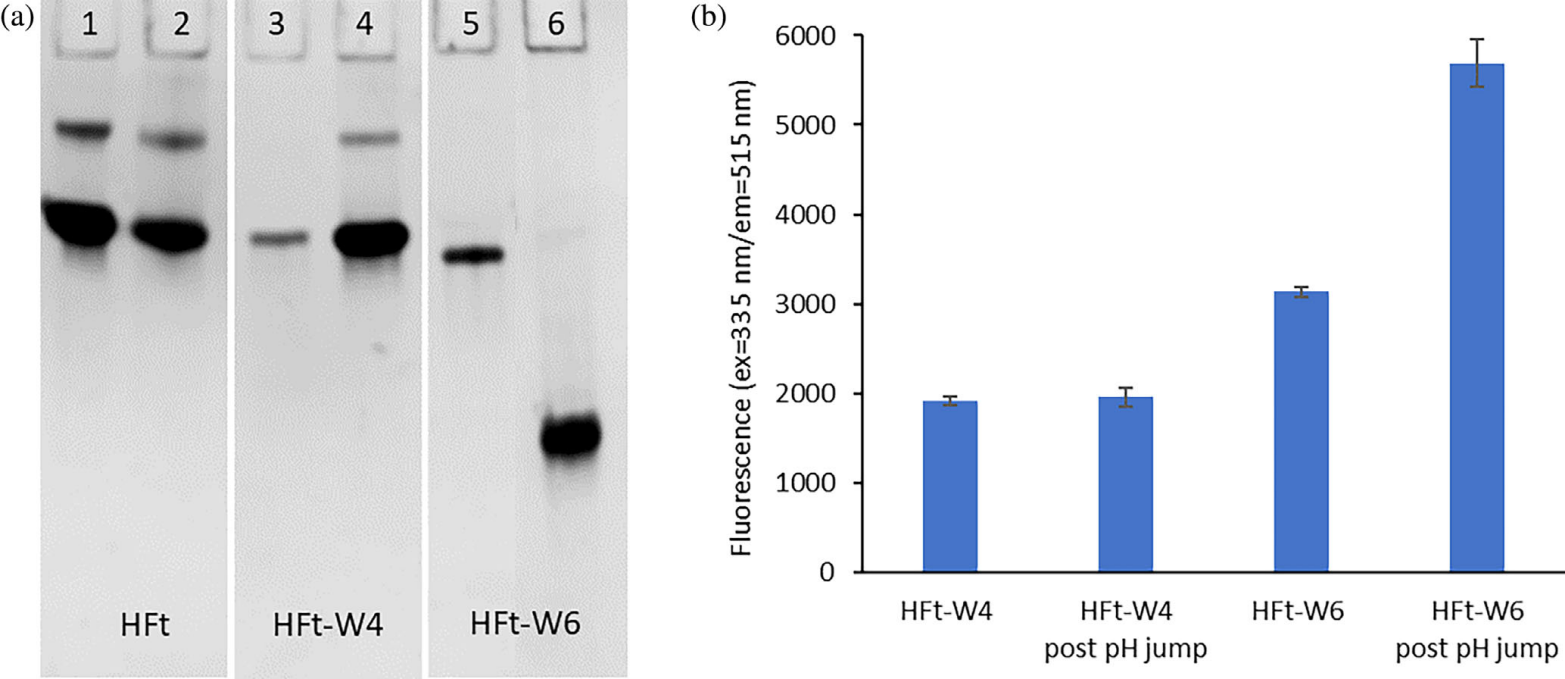
Analysis of human H ferritin, HFt‐W4 and HFt‐W6 in response to the pH jump. (a) Native gel electrophoresis of HFt, HFt‐W4 and HFt‐W6 before (lanes 1, 3, 5) and after (lanes 2, 4, 6) the pH jump. (b) ANS fluorescence of HFt‐W4 and HFt‐W6 mutants before and after the pH jump (data are the mean ± SD of three experiments).

As reported in the literature (Kim et al., 2011; Mohanty et al., 2021), this procedure leads to a significant loss of protein due to the uncomplete reversibility of the process below pH 2.66. To overcome this drawback, we conducted the pH jump under controlled conditions using citrate buffer, ensuring that the pH did not fall below 2.7. Under these conditions, the recovery of correctly reassociated protein was high (>80%) for HFt and HFt‐W4, but not for HFt‐W6. The native gel electrophoresis (Figure 3a) clearly showed a marked difference in electrophoretic mobility for HFt‐W6 after the pH jump, indicating a distinct association state.

To further characterize the association state, we employed the aromatic chromophore ANS, commonly used in the study of protein folding/unfolding processes (Stryer, 1965; Semisotnov et al., 1991). ANS exhibits weak fluorescence in water, but its spectrum undergoes a blue shift, and its intensity increases significantly upon interaction with hydrophobic regions of proteins. Considering that the HFt‐W4 and HFt‐W6 mutants demonstrate a substantial increase in hydrophobic residues within the internal cavity, an incorrect assembly would result in these residues being exposed to the solvent. Consequently, they would become more accessible to ANS interaction. Thus, ANS can serve as a probe to assess the correct quaternary assembly of these mutants. In the case of HFt‐W4, treatment with ANS does not cause an increase in fluorescence following a pH jump, indicating a correctly assembled protein. On the other hand, for HFt‐W6, the fluorescence of ANS significantly increases after the pH jump, indicating that the internal hydrophobic residues are most likely exposed to the solvent due to an incorrect quaternary assembly (Figures 3b and S2).

### 2.2 Drug encapsulation and loaded nanoparticle stability

Given the unsuccessful reassociation of HFt‐W6 into the 24‐mer structure, we exclusively encapsulated drugs in HFt‐W4 and compared it with HFt. As a proof of concept, we used ellipticine, a fluorescent indole alkaloid with multimodal anticancer activity. Indeed, it intercalates DNA, inhibits topoisomerase II causing breaks in the DNA molecule, forms covalent adducts with DNA following activation by cytochrome P450, and acts as an inhibitor of enzymes such as c‐Kit kinase and AKT (Millera and McCarthy, 2012). Despite these interesting properties, the clinical use of this molecule and its derivatives has been limited due to their poor solubility and bioavailability (Dan et al., 2020).

**FIGURE 4:**
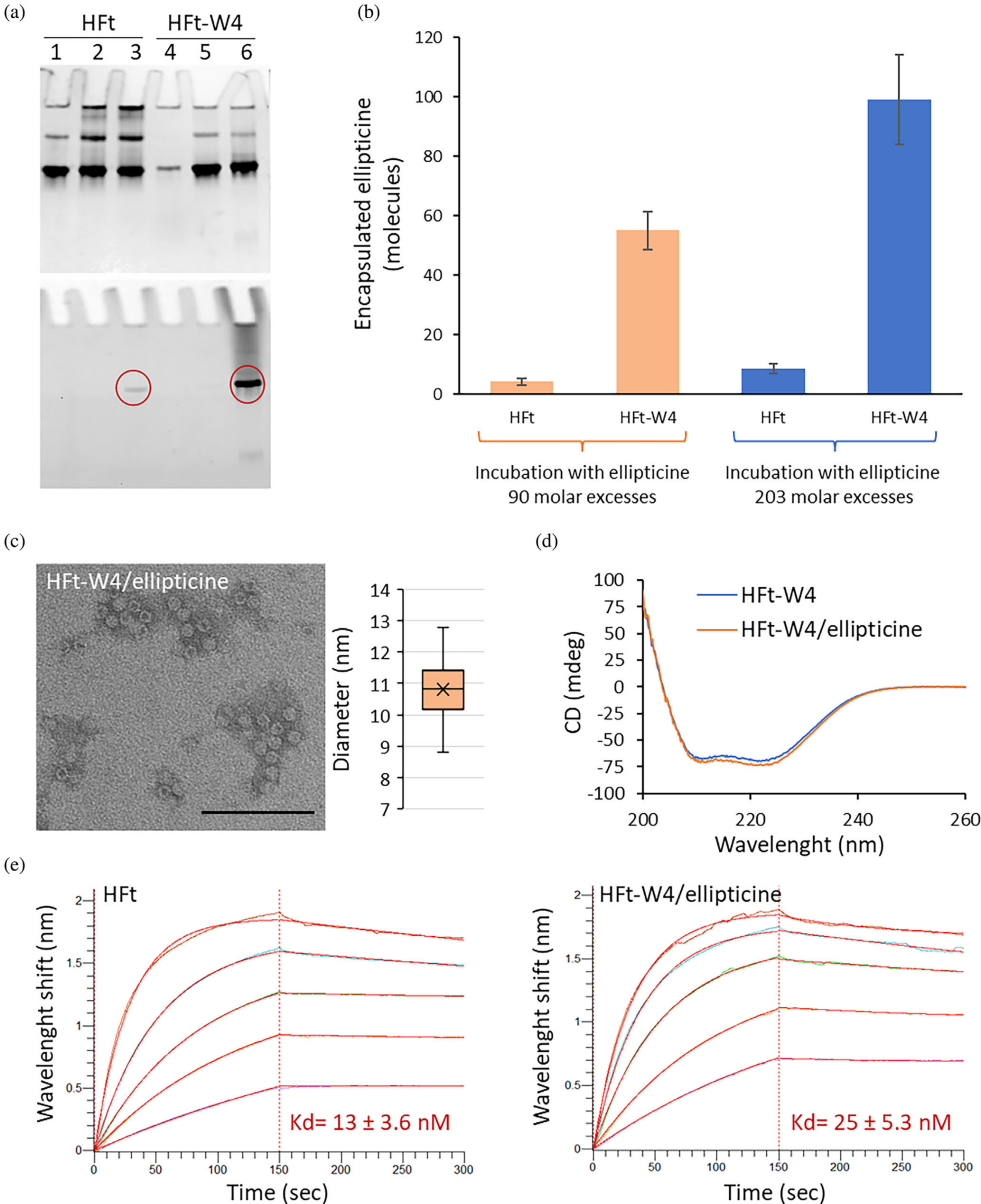
Ellipticine encapsulation. (a) Native gel electrophoresis of HFt and HFt‐W4 before the pH jump (lanes 1, 4), after the pH jump (lanes 2, 5) and after the pH jump in the presence of ellipticine (lanes 3,6). The native gel was stained with Coomassie (upper panel) and analyzed following the ellipticine fluorescence (red circles, lower panel). (b) Effect of ellipticine molar excesses on the encapsulation efficiency (data are expressed as the mean ± SD of three experiments). (c) TEM analysis of HFt‐W4/ellipticine at a protein dilution of 15 μg mL^−1^ (scale bar: 0.1 μm) and the nanoparticle's diameter distribution. (d) CD spectra of HFt‐W4 empty (blue line) or loaded with ellipticine (orange line). (e) BLI recordings representing the binding and release of HFt and HFt‐W4/ellipticine to/from TfR1 immobilized on the sensor chip, together with the Kd constants derived from BLI experiments.

The above‐mentioned properties of HFt‐W4 in terms of high hydrophobicity of the internal cavity and optimal and reversible association/dissociation behavior, make this mutant a good candidate for the encapsulation of this hydrophobic drug and subsequent use for targeted delivery. The acidic pH conditions required for the dissociation of ferritin are also favorable for an increase in the solubility of ellipticine in water (0.153 mg L^−1^, LogP(o/w): 4.8) (Liu et al., 2004) thereby facilitating the encapsulation process. Encapsulation of ellipticine was monitored by native gel electrophoresis (Figure 4a), UV–vis absorption, and HP‐SEC (Figure S3).

Ellipticine was used at 90 and 203‐fold molar excesses with respect to ferritin nanoparticles, and the results are reported in Figure 4b. The best results were obtained using 1 μM ferritin in the presence of 203‐fold excess ellipticine. Due to its limited solubility, even under acidic conditions, testing at higher ratios was not feasible. Under these conditions, as evidenced by TEM and CD analyses (Figure 4c,d), the overall assembly and secondary structure of HFt‐W4 nanoparticles were not affected upon drug loading. In all cases, the HFt‐W4 mutant incorporated a higher number of ellipticine molecules compared to wild‐type HFt, indicating that this type of modification is indeed useful for obtaining nanoparticles suitable for the delivery of hydrophobic molecules. Specifically, the HPLC analysis performed by disassembling the 24‐mer yielded an average number of encapsulated ellipticine molecules equal to 98 for HFt‐W4, almost one order of magnitude higher than the 8 molecules encapsulated by HFt.

These results are also supported by the fluorescence contour maps reported in Appendix S1 in which, characteristically, the presence of ellipticine in the protein cavity leads to the quenching of the tryptophan fluorescence signal (Figure S4).

In view of in vitro cell testing, we have decided to verify whether the ellipticine‐loaded HFt‐W4 nanoparticle maintains its ability to bind to the TfR1 receptor. To achieve this, we utilized biolayer interferometry (BLI), a powerful tool for real‐time measurement of biomolecular interactions. In this experiment, we immobilized his‐tagged TfR1 on a biosensor tip and then immersed it in a solution containing HFt‐W4 loaded with ellipticine, monitoring its binding to the receptor in real‐time and measuring the strength of the interaction (Figure 4e). Our results show a Kd constant of 25 nM, comparable to that of wild‐type HFt (13 nM) obtained under the same experimental conditions.

Building upon the successful encapsulation of ellipticine in the HFt‐W4 mutant, we extended our investigation to include doxorubicin, a widely used antitumor anthracycline drug. This choice was driven by the availability of relevant data on its encapsulation efficiency in HFt. By adopting this comparative approach, we aimed to gain a comprehensive understanding and unbiased evaluation of the encapsulation efficiency exhibited by HFt‐W4. According to the literature, human HFt can encapsulate an average of 20–30 molecules of doxorubicin (Wang et al., 2022; Liang et al., 2014; Bellini et al., 2014; Blazkova et al., 2013; Yin et al., 2022) and our studies confirm these findings (Table S1). Our investigations using the HFt‐W4 mutant revealed the successful encapsulation also of doxorubicin (Figure S5 and Table S1) with an average of 41 encapsulated molecules per protein cage. This result demonstrates the efficient encapsulation capability of HFt‐W4, even for a more hydrophilic molecule such as doxorubicin (logP 1.27, https://www.drugbank.ca/drugs/DB00997), exceeding previous expectations, which suggested limited use for hydrophobic molecules only.

The stability of ferritin nanoparticles loaded with ellipticine or doxorubicin is critical for their potential applications as drug delivery systems. To assess both stability and release profiles of these loaded nanoparticles, we employed HP‐SEC, providing valuable insights into protein cage stability, correct assembly, and drug content. Using HP‐SEC, we can simultaneously evaluate the proper assembly of the protein, which elutes at the typical retention time of the 24‐mer, and the actual drug content estimated by tracking the signals of ellipticine or doxorubicin at the ferritin's retention time. Furthermore, at regular time intervals, the solution containing the loaded nanoparticles was diafiltrated through a 30 kDa concentrator, and the filtrate was analyzed by RP‐HPLC to detect any released molecules of doxorubicin or ellipticine. The absence of a chromatographic signal confirmed that all the cargo is retained within the protein cage and is not released for at least 30 days. The results revealed that the nanoparticles demonstrated excellent stability when stored at 4°C for at least 30 days (or 72 h at 37°C, the experimental conditions used for cell experiments), whether in PBS or culture medium (Figure S6).

This observed stability enhances the feasibility of long‐term storage for the nanoparticle formulation. These findings further support the suitability of ferritin nanoparticles as promising platforms for the controlled delivery of ellipticine and doxorubicin.

### 2.3 In vitro cell experiments

After establishing the efficient loading capacity of the HFt‐W4 mutant for ellipticine and confirming the stability of the resulting complex, as well as its ability to recognize TfR1, our investigation proceeded to evaluate its targeted delivery capabilities. To assess targeted delivery, we selected the HL60 promyelocytic cell line, which is known to overexpress the TfR1 receptor (Daniels et al., 2012; Lyonsa and Pappas, 2019). The overexpression of this receptor on cancer cells, its capacity for internalization, and the essential role of iron in promoting cancer cell proliferation collectively establish this receptor as a readily accessible gateway for drug delivery into malignant cells, making it an attractive target for cancer therapy.

In addition to investigating the delivery of ellipticine, our experimental investigations also included doxorubicin. This decision was made to generate data that can be compared with the existing literature regarding the delivery and efficacy of doxorubicin using ferritin‐based systems.

**FIGURE 5:**
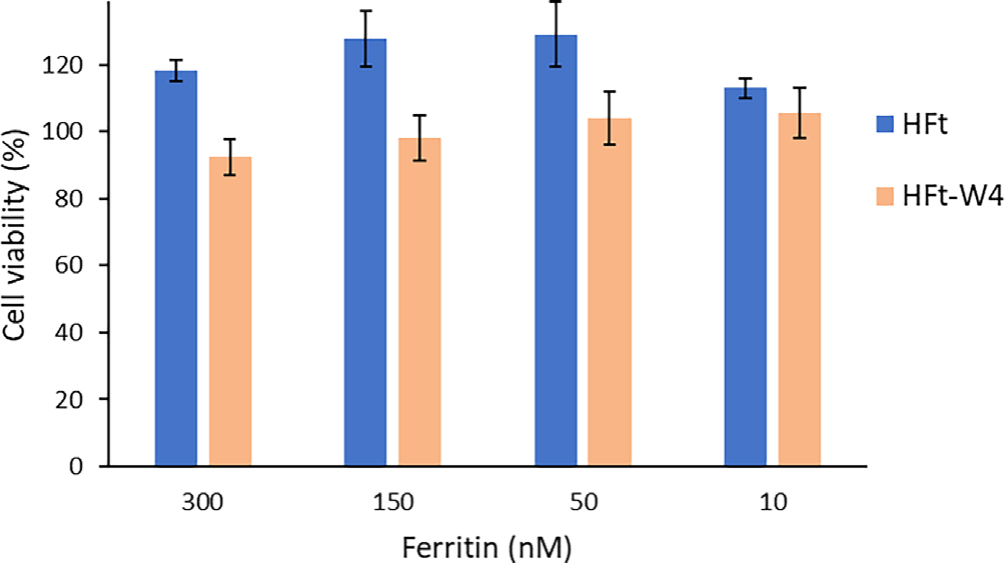
Effect of empty HFt and HFt‐W4 on HL60 viability (mean ± SD, *n* = 3).

The potential cytotoxicity of unloaded ferritin was tested using both HFt and HFt‐W4 at concentrations ranging from 10 to 300 nM over a 72‐h period (Falvo et al., 2016; Mazzucchelli et al., 2017). Figure 5 shows complete absence of cytotoxicity on HL60 cells. By confirming the non‐toxic nature of empty ferritin, we can confidently attribute any observed effects in subsequent experiments to the loaded drug rather than the carrier itself. Ferritin enters HL60 cells through TfR1 (Wu et al., 2023) as blocking this receptor has been shown to reduce its uptake. Considering that the HFt‐W4 mutant does not exhibit any alterations on the external surface and is still able to recognize TfR1 as confirmed by BLI experiments (Figure 4E), it is expected to be internalized in HL60 just as HFt, which is supported by our FACS experiments (Figure S7).

**FIGURE 6:**
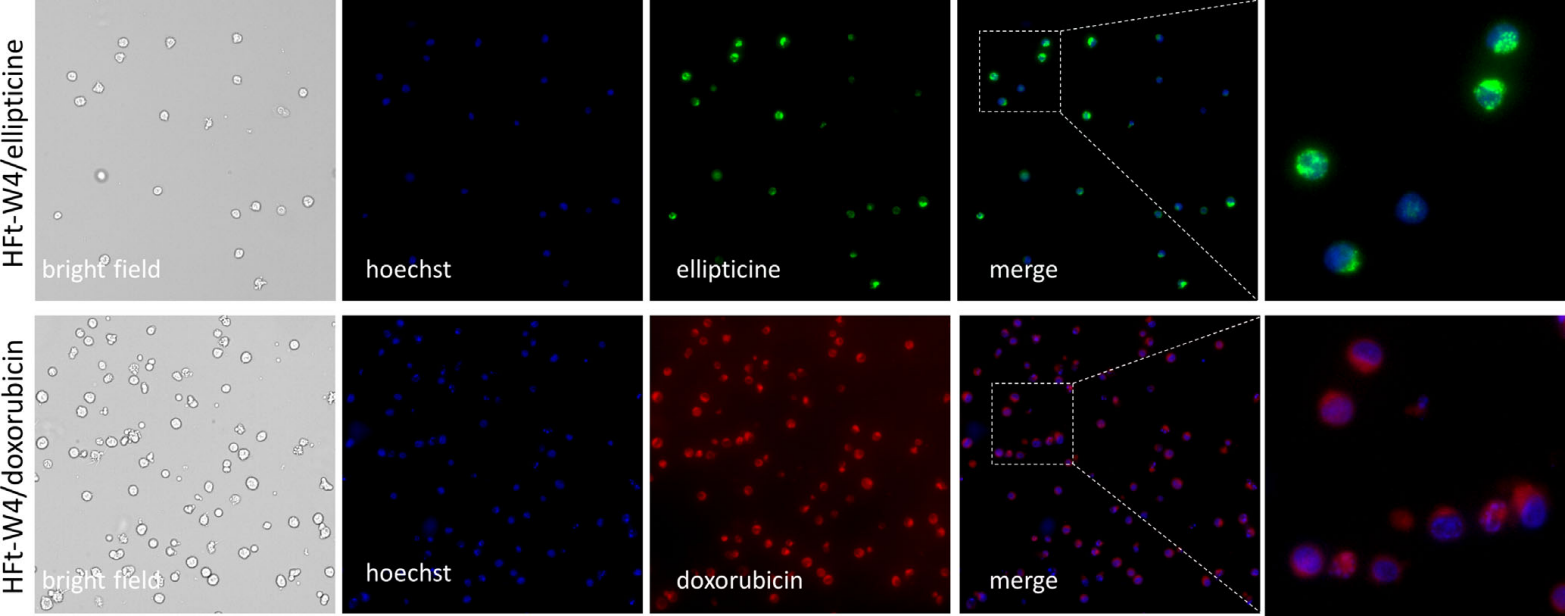
Microscopy analysis of HL60 cells treated with HFt‐W4/ellipticine or HFt‐W4/doxorubicin (24‐h incubation time).

To further validate this hypothesis, we studied the uptake of HFt‐W4 nanoparticles loaded with doxorubicin or ellipticine, both of which possess intrinsic fluorescence. As illustrated in Figure 6, HL60 cells treated with the HFt‐W4 loaded with these drugs exhibited intense green staining (ellipticine) and red staining (doxorubicin) after a 24‐h incubation period.

This intracellular fluorescence can only be attributed to the uptake of the loaded nanoparticles, since purification of the nanoparticles after cargo loading removed any unincorporated drugs. Biochemical characterization confirmed the absence of any traces of the drugs outside the nanoparticles. Furthermore, it was observed that the nanoparticles did not release the drugs for up to 72 h in culture medium at 37°C. These findings imply that the observed cell staining is exclusively attributed to the cargo carried by ferritin.

**FIGURE 7:**
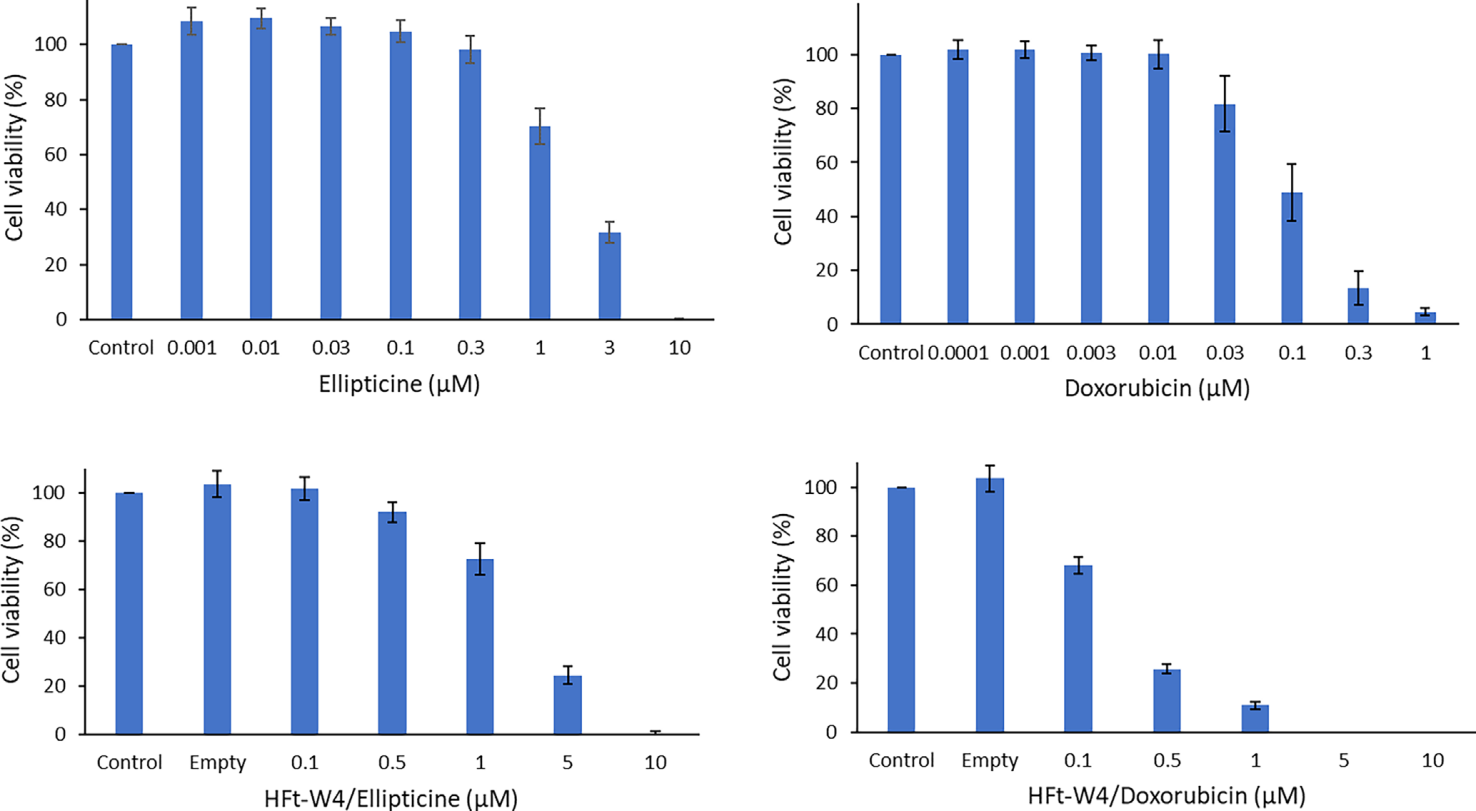
Viability of HL60 cells treated with free ellipticine/doxorubicin or with HFt‐W4 loaded with ellipticine or doxorubicin (mean ± SD, *n* = 4). When conducting experiments with HFt‐W4 loaded with ellipticine or doxorubicin, the specific concentration of the encapsulated drug was taken into account.

HL60 cells were treated with increasing concentrations of ellipticine and doxorubicin to determine the cytotoxicity of these drugs. The MTT assay was used for evaluation of cellular viability. The results confirm that the cells are sensitive to both drugs tested in a dose‐dependent manner (Figure 7). In the experimental setting used, we obtained IC_50_ values of 1.78 ± 0.25 μM for ellipticine and 0.10 ± 0.03 μM for doxorubicin. Both drugs retained comparable cytotoxic properties when carried by HFt‐W4: ellipticine shows an IC_50_ value of 2.05 ± 0.37 μM and doxorubicin 0.19 ± 0.01 μM.

In conclusion, the results of our study highlight the potential of HFt‐W4 as a robust drug delivery system especially designed for the loading of hydrophobic molecules. The enhanced encapsulation capability of this mutant, exemplified by the efficient loading of ellipticine paves the way for its application in delivering hydrophobic therapeutics. In addition, successful encapsulation of doxorubicin in ferritin nanoparticles further supports the versatility of this carrier in accommodating diverse therapeutic compounds. By selectively recognizing the TfR1 receptor, HFt‐W4 can preferentially target tumor cells, thereby limiting potential off‐target effects and reducing toxicity to healthy tissues. This targeted delivery approach may provide benefits such as improved drug accumulation at the site of action, controlled release, and protection against degradation. This assumes importance particularly given that human H ferritin‐based nanoparticles are currently in the preclinical phase for targeted cancer chemotherapy (Falvo et al., 2021) and undergoing clinical trials as a platform for the vaccine development (Zhu et al., 2023).

## 3 MATERIALS AND METHODS

### 3.1 Protein expression and purification

To produce two HFt ferritin mutants (HFt‐W4 and HFt‐W6) with 4 or 6 tryptophan residues that face the internal cavity, synthetic genes were optimized for expression in *Escherichia coli* cells and subcloned into a pET22b vector. Protein expression was induced with 1 mM IPTG at OD_600_ = 0.6 for 16 h at 37°C, after which cells were collected through centrifugation.

The same purification method was used for HFt, HFt‐W4, and HFt‐W6. Bacterial paste from 1 L of culture was sonicated in 50 mL of 20 mM sodium phosphate buffer (pH 7.4) containing 150 mM NaCl and protease inhibitors (Roche). The soluble fraction was then treated with 50% and 70% (NH_4_)_2_SO_4_. The 70% (NH_4_)_2_SO_4_ pellet was suspended in 20 mL of 20 mM sodium phosphate buffer (pH 7.4) containing 150 mM NaCl and extensively dialyzed overnight at 4°C. After dialysis, the protein sample was heat‐treated at 70°C for 10 min. The resulting soluble fraction was then digested with 50 μg mL^−1^ deoxyribonuclease I (Merck) for 1 h at 37°C with the addition of MgCl_2_ (final concentration 2 mM). The protein sample was concentrated to 10 mL and loaded onto a HiLoad 26/600 Superdex 200 pg column that had been previously equilibrated with 20 mM sodium phosphate buffer pH 7.4 containing 150 mM NaCl, using an AKTA‐Pure apparatus (Cytiva). The protein fractions eluting at the elution volume of ferritin (6 fractions, 2 mL each) were combined, concentrated using AmiconUltra‐15 centrifugal filter devices (100 kDa cut‐off), and then sterile filtered and stored at 4°C. The protein concentration was calculated by measuring the UV absorption at 280 nm (HFt *ε*_280_ = 19,000 M^−1^ cm^−1^; HFt‐W4 *ε*_280_ = 40, 910 M^−1^ cm^−1^; HFt‐W6 *ε*_280_ = 50,420 M^−1^ cm^−1^) and protein purity was checked by SDS‐PAGE and High‐Performance Size Exclusion Chromatography (HP‐SEC).

### 3.2 Drug encapsulation

Ellipticine (Merck) and doxorubicin (Merck) encapsulation in HFt and HFt‐W4 was performed according to the following protocol. A solution of 1 μM ferritin 24‐mer in PBS (5 mL) was adjusted to pH 2.7 by adding 0.1 M citrate buffer and 2 M HCl. Next, 0.15 or 0.34 mL of ellipticine stock solution (0.8 mg mL^−1^) in acidic water (1 M HCl/water 1:85) or 1 mL of doxorubicin stock solution (1 mg mL^−1^ in MilliQ water) was added to the protein sample and incubated at room temperature for 15 min. The pH was then adjusted back to 7.4 using 2 M NaOH. Unloaded drug was removed using a PD‐10 desalting column (GE Healthcare Bio‐Science AB, Sweden). The resulting ferritin‐drug nanoparticles were characterized using Native‐PAGE, HPLC, CD spectroscopy, transmission electron microscopy, biolayer interferometry, and fluorescence studies. To measure the doxorubicin content, an extraction step was performed in acidified isopropanol (0.75 M HCl) (Falvo et al., 2016). Doxorubicin was quantified by UV–vis absorption spectroscopy at 485 nm using the molar extinction coefficient *ε* = 12,250 M^−1^ cm^−1^. The same analytical procedure was used to detect the release of doxorubicin analyzing the buffer after diafiltration on Microcon‐30 kDa Centrifugal Filter Unit with Ultracel‐30 membrane (Millipore).

Ellipticine was extracted and quantified by HPLC as described below.

### 3.3 Native‐PAGE

Native electrophoresis analysis was carried out on 4%–15% non‐denaturing acrylamide gel (Mini‐PROTEAN TGX stain‐free, Bio‐Rad) using Tris‐glycine as a running buffer. Electrophoresis was performed at room temperature at a constant voltage of 150–200 V using a Mini‐Protean tetra‐cell electrophoresis apparatus (Bio‐Rad).

### 3.4 High‐performance liquid chromatography

Separations were performed using an Agilent Infinity 1260 HPLC system equipped with both UV and fluorescence detectors to verify the proper assembly of the ferritin nanoparticles after the pH jump and to determine the amount of ellipticine encapsulated.

Analytical size‐exclusion chromatography (HP‐SEC) with an Agilent AdvanceBio SEC 300 Å, 7.8 × 150 mm, 2.7 μm, LC column was employed to verify the ferritin assembly. Isocratic analysis was carried out with phosphate‐buffered saline at pH 7.4 as the mobile phase, with a flow rate of 1.0 mL min^−1^, and UV detection at 220 and 280 nm.

Drug encapsulation, nanoparticle stability and drug release profiles were verified by HP‐SEC following the absorption of ellipticine (427 nm) or doxorubicin (488 nm) at the ferritin elution volume.

To estimate the amount of ellipticine loaded inside ferritin, a Poroshell 120 EC C18 column (3 × 150 mm, 2.7 μm) (Agilent) was used with an isocratic mobile phase of 70% MeOH containing 0.1% TFA, at a flow rate of 0.5 mL min^−1^. The elution of ellipticine was monitored using UV detection at 315 nm. The same analytical procedure was used to detect the release of ellipticine analyzing the buffer after diafiltration on Microcon‐30 kDa Centrifugal Filter Unit with Ultracel‐30 membrane (Millipore).

To extract the encapsulated ellipticine from the ferritin nanoparticles, 25 μL of the ferritin/ellipticine complex (1 μM protein) were heated at 99°C for 5 min, and then 75 μL of methanol was added. The solution was centrifuged for 5 min at 13,000 rpm, and the soluble fraction was analyzed by HPLC (injection: 10 μL). The concentration of ellipticine was calculated using an 8‐points calibration curve ranging from 0.97 to 120 μg mL^−1^ (*y* = 105,646*x* + 47.936, *R* ^2^ = 0.999).

### 3.5 Fluorescence spectroscopy

Fluorescence spectroscopy analysis was performed using a SHIMADZU RF‐6000 spectrofluorophotometer to evaluate both the assembly of HFt, HFt‐W4, and HFt‐W6 and the encapsulation of ellipticine.

The assembly of HFt, HFt‐W4, and HFt‐W6 was analyzed before and after the pH jump using 8‐anilinonaphthalene‐1‐sulfonic acid (ANS) (Merck) as a probe, which interacts with the hydrophobic residues exposed to the solvent. To perform this analysis, 30 μM ANS was added to 1 μM protein sample (3 mL), and the fluorescence was recorded at *λ*_ex_ = 335 nm and *λ*_em_ = 515 nm.

To record the fluorescence of free and encapsulated ellipticine, the spectrofluorophotometer was set at *λ*_ex_ = 429 nm and *λ*_em_ = 534 nm. 3D fluorescence maps of empty and ellipticine‐loaded ferritin nanoparticles were acquired in the emission range 270–600 nm upon excitation in the range 270–550 nm.

### 3.6 Transmission electron microscopy negative staining

Protein solutions (15 μg mL^−1^) were diluted 1:10 in buffer and the drop‐on‐grid method was used for analysis: 5 μL of each sample were placed onto formvar–carbon coated grids, and gently blotted with filter paper after 5 min. The samples were then stained using a 4% ammonium molybdate (Merck) solution for 30 s, followed by another blotting with filter paper and air drying. The grids were examined at 100 kV using an EM208S transmission electron microscope (TEM) (FEI—Thermo Fisher Scientific in Eindhoven, The Netherlands), equipped with the Megaview II SIS camera (Olympus‐SIS, Milan, Italy).

To determine the ferritin diameter size distribution, high magnification micrographs were analyzed using the Image J software (version 1.29, NIH, Bethesda, MD). Manual measurements of more than 100 particles were performed for each sample, and the diameter size distributions were calculated using Microsoft Excel 2016 software.

### 3.7 Kinetics of iron oxidation

The kinetics of iron oxidation were investigated through a kinetic analysis of iron loading into apoferritin under aerobic conditions using a Jasco spectrophotometer. A 10 mM Fe^2+^ solution was prepared by dissolving Fe(NH_4_)_2_(SO_4_)_2_·6H_2_O (Merck) in 1 mM HCl. The iron loading reaction was initiated by adding 25 μL of 10 mM Fe^2+^ to 2 mL of 0.3 μM apoferritin in 50 mM HEPES buffer at pH 7.4. Iron oxidation was monitored by following the absorbance at 310 nm. Additionally, iron autoxidation was monitored under the same conditions as described above.

### 3.8 Biolayer interferometry

The interactions between TfR1 and HFt or HFt‐W4 loaded with ellipticine were measured through biolayer interferometry (BLI) using the Octet N2 system (Sartorius). His‐tagged TfR1 (50 μg mL^−1^) (Merck) was immobilized on nickel nitriloacetic acid (Ni‐NTA) biosensors (Sartorius) according to the Sartorius biosensors' datasheets. Subsequently, various concentrations of HFt (ranging from 25 to 500 nM) or HFt‐W4 loaded with ellipticine (ranging from 50 to 400 nM) were added, and the association/dissociation kinetics were recorded. The data were analyzed using the Octet software to extrapolate the kinetic parameters. All association and dissociation curves were fitted using a single exponential function.

### 3.9 Circular dichroism

The secondary structure of HFt, HFt‐W4, HFt‐W6, and ellipticine‐loaded HFt‐W4 was assessed using far‐UV circular dichroism (CD) analysis. CD spectra were recorded between 200 and 260 nm in a 0.1 cm quartz cuvette, using a Jasco J‐815 spectropolarimeter (Jasco) with ferritin samples at a concentration of 0.5 mg mL^−1^ in 20 mM phosphate buffer at pH 7.4. Data collection and analysis were performed using the software package Spectra Manager (Jasco).

### 3.10 Cell culture

HL60 cell line (ATCC) was cultured in RPMI 1640 medium supplemented with 10% fetal bovine serum and 1% penicillin/streptomycin solution in 75 cm^2^ culture flasks at 37°C in a humidified atmosphere of 5% CO_2_.

### 3.11 Cell viability assay

The effect of ellipticine, doxorubicin, ferritin (HFt and HFt‐W4), and HFt‐W4 loaded with ellipticine or doxorubicin was determined on HL60 cell line by MTT assay.

Ellipticine stock solution was prepared in DMSO at a final concentration of 10 mM whereas doxorubicin was dissolved in MilliQ water at a final concentration of 3.68 mM.

HL60 cells were seeded in a 96‐well microplate at a density of 1 × 10^4^ cells per well and treated with various concentrations of free ellipticine (1.0 nM to 10 μM final concentration) or doxorubicin (0.1 nM to 1.0 μM final concentration), as well as empty HFt and HFt‐W4 (50–300 nM final concentration). When conducting experiments with HFt‐W4 loaded with ellipticine or doxorubicin, the experimental setup took into account the specific concentration of the encapsulated drug.

After 72 h incubation (37°C, 5% CO_2_), the MTT solution (3 mg mL^−1^ in PBS) was added, the microplates were incubated for 2 h at 37°C and the cells were lysed using a solution composed of 0.1 M HCl and 5% Triton X‐100 in isopropanol. The absorbance at 570 nm was measured for each well by microplate readers Tecan Infinite M 200 (Life Sciences). The absorbance at 690 nm and the mean absorbance of cell‐free controls was subtracted as a background. The viability of the control cells was set as 100%, and the values of the treated cells were normalized accordingly. IC_50_ was defined as the concentration required to inhibit cellular viability to 50% of the drug‐free control and was calculated using the GraphPad Prism software.

### 3.12 Flow cytometry

The uptake of FITC‐labeled ferritin was assessed by flow cytometry (Sony Spectra cell analyzer SA3800). HL60 cells were seeded at 1 × 10^5^ per well in a 96‐well microplate and incubated for 24 h at 37°C in a 5% CO_2_‐saturated atmosphere with 50, 150, and 300 nM of empty HFt or HFt‐W4. For each experimental point, 1 x 10^4^ events were taken, and the data were analyzed by the FCS Express software.

### 3.13 Microscopy

Microscopy analyses were performed using a Nikon Eclipse Ti inverted epifluorescence microscope. HL60 cells were seeded at 7 x 10^5^ per well in a 24‐well plate and incubated with HFt‐W4/ellipticine or HFt‐W4/doxorubicin for 2, 6, and 24 h. Cells were then washed twice with PBS. Nuclei were stained with Hoechst 33342 (10 μg mL^−1^ in PBS) and then washed twice with PBS before the microscopy analysis. Signals from the different fluorescent probes (Hoechst and ellipticine or doxorubicin) were taken in sequential scanning mode to avoid spectral overlap (false‐positive signal) and with matching exposure setting between experimental groups.

## AUTHOR CONTRIBUTIONS

Conceptualization: Alberto Macone, Alessandra Bonamore; Methodology: Alessio Incocciati, Jan Kubeš, Roberta Piacentini; Investigation: Alessio Incocciati, Jan Kubeš, Roberta Piacentini, Chiara Cappelletti, Sofia Botta, Lucia Bertuccini; Validation: Tomáš Šimůnek, Alberto Macone, Alessandra Bonamore; Formal analysis: Alessio Incocciati, Jan Kubeš; Supervision: Tomáš Šimůnek, Alberto Macone, Alessandra Bonamore; Funding acquisition: Alberto Boffi, Alberto Macone, Jan Kubeš; Visualization: Alessio Incocciati, Alberto Macone, Alessandra Bonamore; Project administration: Tomáš Šimůnek, Alberto Boffi, Alberto Macone, Alessandra Bonamore; Writing ‐ original draft: Alberto Macone, Alessandra Bonamore; Writing ‐ review & editing: Tomáš Šimůnek, Alberto Boffi, Alberto Macone, Alessandra Bonamore.

## CONFLICT OF INTEREST STATEMENT

The authors declare no conflict of interest.

## Supporting information


**FIGURE S1:** Kinetics of iron loading into apoferritin (HFt) and its mutants, HFt‐W4 and HFt‐W6.
**FIGURE S2:** Fluorescence contour maps of HFt‐W4 and HFt‐W6 treated with ANS before and after the pH jump.
**FIGURE S3:** Encapsulation of ellipticine in HFt‐W4 mutant monitored by UV–vis and HP‐SEC.
**FIGURE S4:** Fluorescence contour maps of HFt and HFt‐W4 empty or loaded with ellipticine.
**FIGURE S5:** HP‐SEC analysis of HFT‐W4 loaded with doxorubicin.
**FIGURE S6:** Stability of HFt‐W4 loaded with ellipticine or doxorubicin.
**FIGURE S7:** FACS analysis of HL60 cells incubated for 24 h with FITC‐labeled HFt and FITC‐labeled HFt‐W4.
**TABLE S1:** Doxorubicin molecules encapsulated within HFt and HFt‐W4.
