# Supplementary material for: Hydrophobicity‐enhanced ferritin nanoparticles for efficient encapsulation and targeted delivery of hydrophobic drugs to tumor cells
Source: Protein Sci. 2023 Dec 1;32(12):e4819. doi: 10.1002/pro.4819 (PMC10661074; doi:10.1002/pro.4819)
Supplement: Supplementary file 1 — FIGURE S1: Kinetics of iron loading into apoferritin (HFt) and its mutants, HFt‐W4 and HFt‐W6. FIGURE S2: Fluorescence contour maps of HFt‐W4 and HFt‐W6 treated with ANS before and after the pH jump. FIGURE S3: Encapsulation of ellipticine in HFt‐W4 mutant monitored by UV–vis and HP‐SEC. FIGURE S4: Fluorescence contour maps of HFt and HFt‐W4 empty or loaded with ellipticine. FIGURE S5: HP‐SEC analysis of HFT‐W4 loaded with doxorubicin. FIGURE S6: Stability of HFt‐W4 loaded with ellipticine or doxorubicin. FIGURE S7: FACS analysis of HL60 cells incubated for 24 h with FITC‐labeled HFt and FITC‐labeled HFt‐W4. TABLE S1: Doxorubicin molecules encapsulated within HFt and HFt‐W4. [file PRO-32-e4819-s001.docx]

**SUPPLEMENTARY MATERIAL**

**Hydrophobicity-Enhanced Ferritin Nanoparticles for Efficient Encapsulation and Targeted Delivery of Hydrophobic Drugs to Tumor Cells**

Alessio Incocciati^1^, Jan Kubeš^2^, Roberta Piacentini^1,3^, Chiara Cappelletti^1^, Sofia Botta^1^, Lucia Bertuccini^4^, Tomáš Šimůnek^2^, Alberto Boffi^1^, Alberto Macone^1*^, Alessandra Bonamore^1^

^1^Department of Biochemical Sciences “A. Rossi Fanelli”, Sapienza University of Rome, Piazzale Aldo Moro 5, Rome 00185, Italy

^2^Department of Biochemical Sciences, Faculty of Pharmacy in Hradec Králové, Charles University, Akademika Heyrovského 1203, 500 03 Hradec Králové, Czech Republic

^3^Center of Life Nano- & Neuro-Science, Italian Institute of Technology, Viale Regina Elena 291, 00181 Rome, Italy

^4^ Core Facilities, Istituto Superiore di Sanità, Viale Regina Elena 299, 00161 Rome, Italy

*Corresponding author: Alberto Macone (alberto.macone@uniroma1.it)

**CONTENT**

**Figure S1:** Kinetics of iron loading into apoferritin (HFt) and its mutants, HFt-W4 and HFt-W6.

**Figure S2:** Fluorescence contour maps of HFt-W4 and HFt-W6 treated with ANS before and after the pH jump

**Figure S3:** Encapsulation of ellipticine in HFt-W4 mutant monitored by UV-vis and HP-SEC

**Figure S4:** Fluorescence contour maps of HFt and HFt-W4 empty or loaded with ellipticine

**Figure S5:** HP-SEC analysis of HFT-W4 loaded with doxorubicin

**Figure S6:** Stability of HFt-W4 loaded with ellipticine or doxorubicin

**Figure S7:** FACS analysis of HL60 cells incubated for 24 h with FITC-labelled HFt and FITC-labelled HFt-W4

**Table S1:** Doxorubicin molecules encapsulated within HFt and HFt-W4

Figure S1. Kinetics of iron loading into apoferritin (HFt) and its mutants, HFt-W4 and HFt-W6. The reaction was initiated by adding 25 µL of 10 mM Fe^2+^ to 0.3 µM apoferritin (at a ratio of 400:1 iron/protein cage) and monitoring the signal at 310 nm. The experiment was conducted under aerobic conditions, and to account for iron autoxidation, a reaction blank was carried out by adding iron to the reaction buffer (shown as the blue line). It was observed that iron autoxidation occurred at a slower rate compared to the ferritin-catalyzed reaction.

Figure S2. Fluorescence contour maps of HFt-W4 and HFt-W6 treated with ANS before and after the pH jump. For the HFt-W6 mutant, the fluorescence of ANS significantly increases after the pH jump, indicating that the internal hydrophobic residues are exposed to the solvent due to an incorrect quaternary assembly

Figure S3. Encapsulation of ellipticine in HFt-W4 mutant monitored by UV-vis (left) and HP-SEC (right). HP-SEC analysis shows the absorbance of ellipticine (427 nm) at the elution volume of HFt-W4 followed at 280 nm

Figure S4. Fluorescence contour maps (excitation: 270 nm - 550 nm; emission: 270 nm - 600 nm) of HFt and HFt-W4 empty or loaded with ellipticine. The presence of ellipticine in the protein cavity leads to the quenching of the tryptophan fluorescence signal.

Figure S5. HP-SEC analysis of HFT-W4 loaded with doxorubicin. The chromatogram shows the absorbance of doxorubicin (488 nm) at the elution volume of HFt-W4 followed at 280 nm.

Figure S6. Stability of HFt-W4 loaded with ellipticine or doxorubicin. The stability of the nanoparticle loaded with drugs was assessed through HP-SEC analyses monitoring the absorbance of ellipticine (427 nm) and doxorubicin (488 nm) at the elution volume of HFt-W4. The loaded nanoparticles showed excellent stability when stored at 4°C for at least 30 days, whether in PBS or culture medium.

Figure S7. FACS analysis of HL60 cells incubated for 24 h with FITC-labelled HFt and FITC-labelled HFt-W4 in the concentration range 50 nM – 300 nM.

|  | **starting ferritin**  **concentration** | **doxorubicin**  **molar excess** | **Encapsulated**  **doxorubicin molecules** |
| --- | --- | --- | --- |
| **HFt** | 1 µM | 344 | 22.33 ± 4.04 |
| **HFt-W4** | 1 µM | 344 | 41.66 ± 3.51 |

Table S1. Doxorubicin molecules encapsulated within HFt and HFt-W4 (mean ± SD, n=3)
